# Study on the Flow, Foaming Characteristics and Structural Strength of Polypropylene Structural Foam Injection Molding by Innovative Nitrogen and Molten Plastic Mixing Mechanism

**DOI:** 10.3390/polym15092116

**Published:** 2023-04-28

**Authors:** Po-Wei Huang, Hsin-Shu Peng, Sheng-Jye Hwang, Chao-Tsai Huang

**Affiliations:** 1 Program of Mechanical and Aeronautical Engineering, Feng Chia University of Engineering and Science, Taichung 407102, Taiwan; bowei8915@gmail.com; 2 Department of Mechanical and Computer Aided Engineering, Feng Chia University of Engineering and Science, Taichung 407102, Taiwan; 3 Department of Mechanical Engineering, National Cheng Kung University of Engineering, Tainan 70101, Taiwan; jimppl@mail.ncku.edu.tw; 4 Department of Chemical and Materials Engineering, Tamkang University of Engineering, New Taipei City 251301, Taiwan; cthuang@mail.tku.edu.tw

**Keywords:** structural foam injection molding technology, foaming process-sensing system, polypropylene, melt rheology, viscosity index

## Abstract

Plastic foam molding methods include thermoforming, extrusion and injection molding. Injection foam molding is a one-time molding method with high production efficiency and good product quality. It is suitable for foamed plastic products with complex shapes and strict size requirements. It is also the main method for producing structural bubbles. In this investigation, we developed a structural foam injection molding technology using the gas supply equipment connected to the unique plasticizing mechanism of the injection machine and studied its influence on the specimens’ melt rheology quality and foam structures. In the experiment, the forming material was polypropylene (PP), and the gas for mixing/forming foaming characteristics was nitrogen (N_2_). Additionally, in order to observe the rheological properties of N_2_/melt mixing, a melt flow specimen mold cavity was designed and the change in the melt viscosity index was observed using a melt pressure sensing element installed at the nozzle position. With the nitrogen supply equipment connected to a unique plasticizing mechanism, the mixing of gas and molten plastic can be achieved at the screw plasticizing stage, where the foaming effect is realized during the melt-filling process due to the thermodynamic instability of the gas. It was also found that an increase in N_2_ fill content increased melt fluidity, and the trend of melt pressure and melt viscosity index showed that the higher the gas content, the lower the trend. The foaming characteristic depends on the gas thermodynamic instability and the pressure release, so it can be seen from the melt fill path that, the greater the pressure near the gate, the lower the foaming amount and the internal structure (SEM) after molding; the farther from the gate, the greater the relative increase in the foaming growth/amount. This phenomenon will be more obvious when the N_2_ fill content is increased.

## 1. Introduction

Driven by the awareness of lightweight and environmental protection goals, in addition to choosing alternative materials or adopting new technologies to achieve lightweight goals, enterprises must also consider the adverse impact of materials on the environment. Therefore, “foam molding” has become an important development application that has attracted much attention. The technology of physical foaming is becoming more and more mature, and the desired result can be achieved by adjusting the process parameters, and gradually replacing the chemical foaming process. Microcellular polymer foams are a kind of advanced polymeric foam that shows lightweight and enhanced thermally insulating performance compared with traditional polymer foams [1]. Therefore, the lightweight process of major products is extended; polymeric foams can have enhanced mechanical [2,3], thermal, and acoustic [4,5] properties, as well as significant reductions in weight in comparison to their solid counterparts, owing to the porous nature and cellular structure of the material. This combination of properties has seen cellular foams incorporated into several areas, such as automotive parts, food packaging, and insulation. As such, researchers have sought to produce foams from an increasing range of polymers [6,7]. Since the development of polymer microcellular foams, numerous studies have been conducted on foaming behavior [8,9,10] and cell structure control [11,12,13,14]. For example, the morphology and compression properties of such foams were detailed through study, and it was shown that the nucleation and directional growth of the cells are promoted by the introduction of the multi-layer interface into the polymer matrix. When the distance of the multi-layer interface is smaller than the critical nucleation size of the cell, the microcellular foams with uniform, continuous, and directionally multi-layer cell structures can be prepared, and the unit cell shape is well controlled [14]. Herein, 10-fold expansion ratio foams were successfully fabricated for the first time by a foam injection molding (FIM) technique using a newly developed polypropylene (PP). Different rheological curves were investigated to distinguish the strain-hardening behavior and melt strength of the PP; this study revealed that the introduction of long-chain branches is a good approach to fabricating high-expansion microcellular foams for different potential applications, such as construction and transportation [15,16]. In addition to this, the crystallization and foaming behaviors of a semi-crystalline polymer (PS) in conditions comparable to those found in polymer processing, where the polymer melt experiences shear under elevated pressures, are key for modeling polymer processes and predicting the final structure and mechanical properties of polymer products [17,18]. Among them, a significant amount of research dedicated to improving thermoplastic foam ability is focused on the melt fluidity and molecular weight distribution of the finished product [19]. The cell structure significantly affects the properties of polymer foams and determines their final applications. Therefore, cell structure control has long been an important subject in both academia and industry.

In the traditional injection molding process, the weight of a molded product is related to its quality, meaning that consistency in the weight of items can be used as an indicator of consistency in product quality. The pressure-volume-temperature (P-V-T) relationship in the melt dictates that inconsistencies in the specific volume can be attributed to variations in pressure and temperature. Difficulties in measuring the specific volume in injection molding processes prompted us to develop the viscosity index (VI), proposed in the literature [20,21], to represent the relationship between the weight of a molded item and its material properties. In addition, quality indices can help to rapidly determine the quality of the finish without the need for any measuring equipment, a process that is feasible for quality control in actual injection molding operations. Although injection-molded finished products are not without defects, their quality has improved considerably because of the aforementioned advancements. For example, plastic melts are shear viscous and the volume flow rate has a large influence on the resulting viscosity. For this reason, the characteristic flow resistance is furthermore scaled to the mean of the injection speed. To characterize the quality of the melt, this normalized characteristic parameter, the viscosity index (VI), can be used [22]. Therefore, the calculation formula (Equation (1); [23,24,25]) of a viscosity index was introduced to compare the effects of the melt mixed with or without gas, and the gas content changes. During structural foam injection molding, properties of the melt rheology quality can vary considerably due to the difference between gas content, melt pressure and foam structure. The material properties during the injection molding process were characterized by formulating a VI.

(1) $$VI_{Injection}=\int_{t=point\;2}^{t=point\;1}P_{Melt}\left(t\right)dt$$

“VI” refers to the melt viscosity index, “trigger-point” refers to the position point of screw advance and time calculation (“point 1” is when the melt contacts the pressure sensor, and the filling start time (*t*); “point 2” is when the melt reaches the V/P switch-over position and expresses the filling time end (t)), and $P_{Melt}$ refers to the melt flow pressure. Moreover, the FIM for foaming is initiated by the gas release, compatibility with melt, and nucleation of bubbles [26,27,28]; each bubble draws gas from its surrounding melt in a diffusive process. If the gas depletion is not uniform, the melt gas content initially drops much more near the bubble/melt interface than from the bulk of the surrounding melt. This no-uniform gas depletion results in a radial gas concentration profile around each bubble, where the minimum gas concentration is at the bubble/melt interface; at the same time, the viscosity of a polymer melt strongly depends on gas content, and its melt viscosity decreases with increasing gas content [28,29,30].

Therefore, a mechanism for mixing nitrogen and molten plastic was designed by the authors to observe the melt-filling/porous foaming behavior of thermoplastics through practical experiments in this study. Unlike the common thermoforming, extrusion and injection molding, and supercritical fluid foaming processes, this study is a novel design that combines a conventional gas-assisted device with the injection barrel mechanism of an injection molding machine. Gas-permeable steel is embedded in the neck of the screw tips for gas release and the gas-assisted equipment is used as the gas supply source. The foaming melt (molten plastic with dispersed nitrogen) is formed by mixing the gas released from the permeable steel with the molten plastic. An experiment was also conducted to evaluate the flow, foaming characteristics, and structural strength of polypropylene structural foam injection molding with an innovative nitrogen and molten plastic mixing mechanism.

## 2. Model, Materials, and Methods

### 2.1. Molding Mechanism

#### 2.1.1. Injection Molding Machine

**Figure 1:**
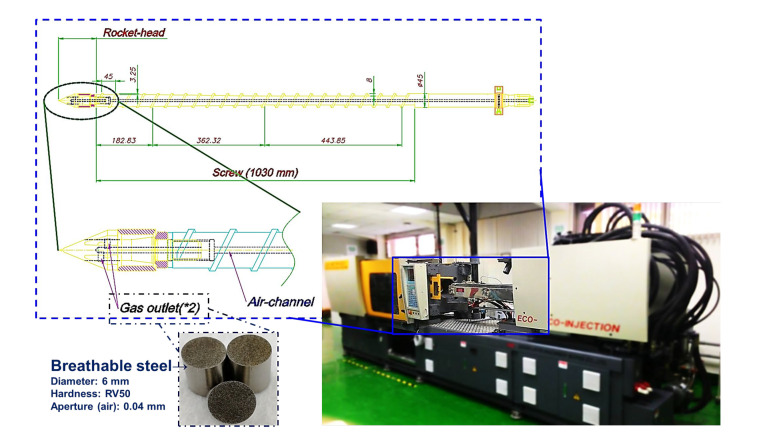
Appearance of structural foam injection molding machine and screw design with breathable steel location.

**Figure 2:**
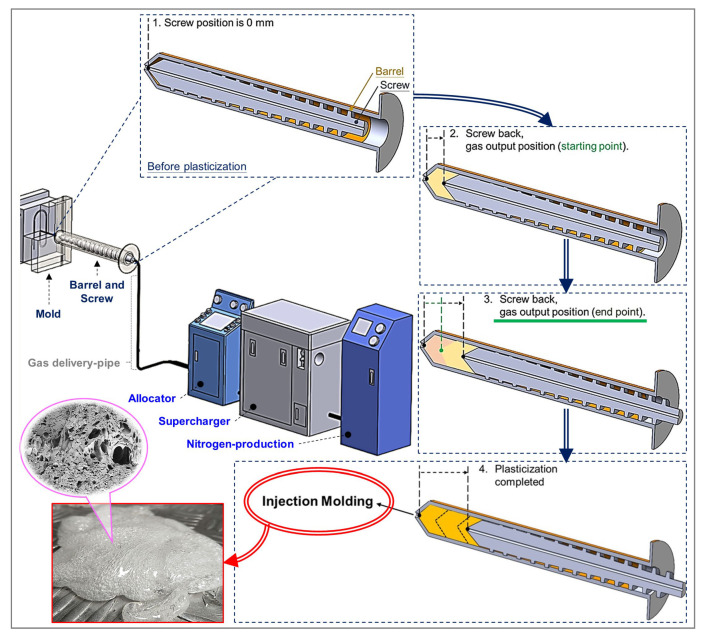
Flow diagram of plasticization, filling, and packing processes in the structural foam injection mechanism.

The structural foam injection machine (basic model CLF-180-TXR-ECO) used in this study was developed jointly with CLF (Chuan-Lih-Fa Machinery Works Co Ltd.; Tainan City, Guanmiao District, Taiwan), with a total clamp tonnage of 180 tons, a maximum injection speed of 142 mm/s, an ECO STAR servo system of 16 kW, and an injection pressure maximum set value of 140 bar. In order to explore and observe the physical foaming mechanism and the compatibility state of its gas and melt, a plasticizing/injection unit with a gas foaming mechanism design was applied (Figure 1), comprising (i) a screw with a hollow structure and gas-output channel, (ii) a screw rocket head with breathable steel elements, and (iii) a screw-end connected to conventional gas-assisted equipment. In this study, the screw is characterized as a hollow design, and the end of the screw mechanism is connected to a gas channel and a gas assist device. Gas-permeable steel is embedded in the neck of the screw tips for gas release and the gas-assisted equipment is used as the gas supply source. The foaming melt (molten plastic with dispersed nitrogen) is formed by mixing the gas released from the permeable steel with the molten plastic (Figure 2 is the plasticizing period of the machine and the gas output into the barrel, as well as the mixing process; (1) the initial position of the screw is 0; (2) the screw position is back according to the parameter setting and back up to the starting position of gas addition; (3) the gas is added to the end position of the parameter setting as the screw moves backward; and (4) the screw retracts to the parameterized end position).

For related parameter settings, refer to Table 1. The temperature profile along the barrel was 230 °C (melt temperature), the injection pressure setting was 140 bar, the injection time was 1.5 s, the injection speed was 70 mm/s and cooling time was 30 s, the mold temperature was 60 °C, the screw speed was 60 rpm, the volume was fixed by a screw stroke of 30 mm, and V/P switch-over position was 1 mm, and without back pressure (these parameters were defined as the standard/base process).

#### 2.1.2. Nitrogen Device

**Figure 3:**
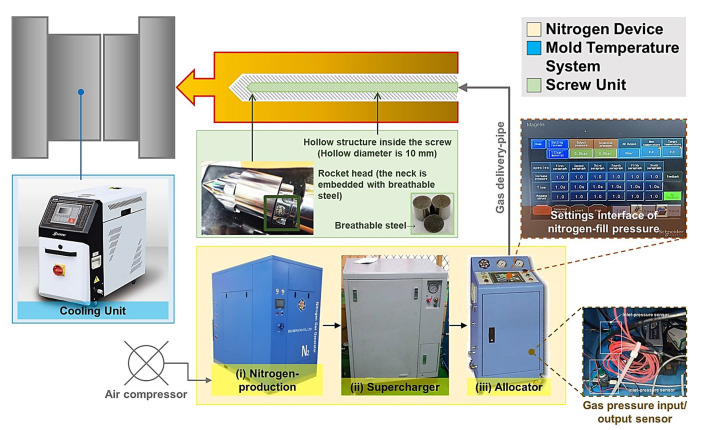
Structure diagram of injection machine with nitrogen supply equipment connected to a unique plasticizing mechanism.

The nitrogen device and its modular system used in this study were developed jointly with DIAMONANO (Ding-Cheng Nano Enterprise Co., Ltd.; Changhua City, Changhua County, Taiwan), as shown in Figure 3. The modularized system includes (i) a nitrogen-producing mechanism that connects the air from the air compressor and filters and extracts the nitrogen, (ii) a booster for compressing and storing gas, and (iii) a distributor for setting the gas content.

#### 2.1.3. Preparing the Specimens

**Figure 4:**
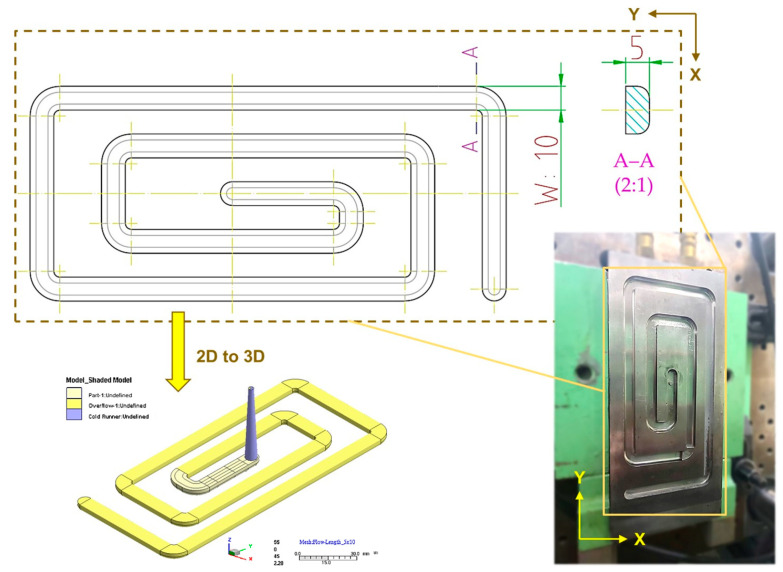
Design and appearance of melt flow specimens (10 mm × 5 mm (W × H)), and schematic of mold cavity.

To mold the specimens of the PP with different gas-fill contents, and for the experiment to investigate the material-flow variation experiment, a spiral-flow mold design was used (Figure 4; specimen size is 1000 mm (total length), 10 mm (width), 5 mm (thickness)).

### 2.2. Molding and Foaming Materials

The experiment and molding material used was polypropylene (PP, reference PT231), which was fabricated using an injection molding process by using LCY GROUP (Taipei City, Songshan District, Taiwan), and the PP melt index was 24 g/10 min.

### 2.3. Characterizations

Through this structure and the execution of the experiment, (i) the compatibility between nitrogen and the melt, (ii) the pressure trend of the melt during the filling stage after compatibility, and (iii) the introduction of the equation (Equation (1) calculates the viscosity index) discuss the viscosity index and observe the changes in its fluidity; (iv) the impact of the pressure changes within the melt on the foaming structure and density; and (v) the impact of comparative melt fluidity and cell density on the structural integrity of the end product.

- For the analysis and experimentation of melt rheology, 30 specimens were tested under the same molding parameters. The last 20 test molded samples’ average value was used for melt flow pressure measurement, calculation of the melt-viscosity index, and observation of foaming shape.
- Carried out with different gas contents (“wt%” is the percentage by weight (%); wt (weight)), and according to the change of its content, the melt pressure/viscosity index, and foaming characteristics, the appropriate comparative trends were explored and the parameter changes carried out in the second stage.
- In the second stage, the changes in the effect of cell density on the structural strength of PP were evaluated by varying several parameter settings of the process (screw speed, melt temperature, injection speed, etc.; Table 2).

#### 2.3.1. Measurement of Melt Flow Pressure

**Figure 5:**
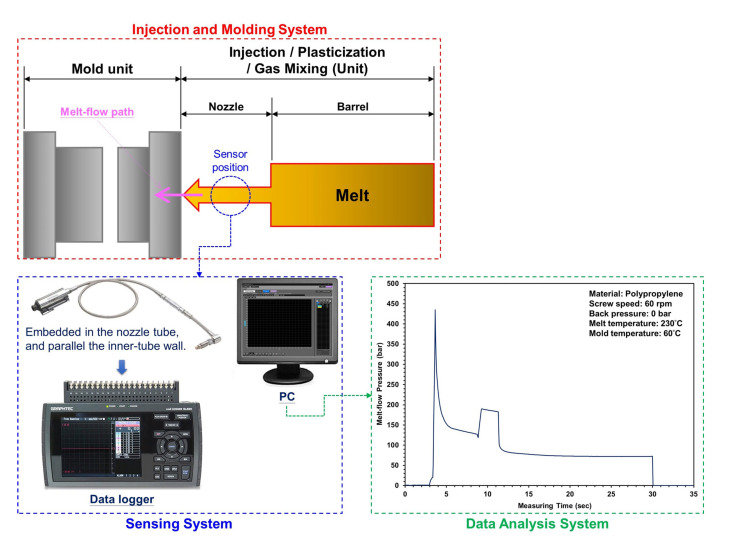
Schematic of sensor locations and sensing system and the melt flow pressure trend with nozzle.

**Figure 6:**
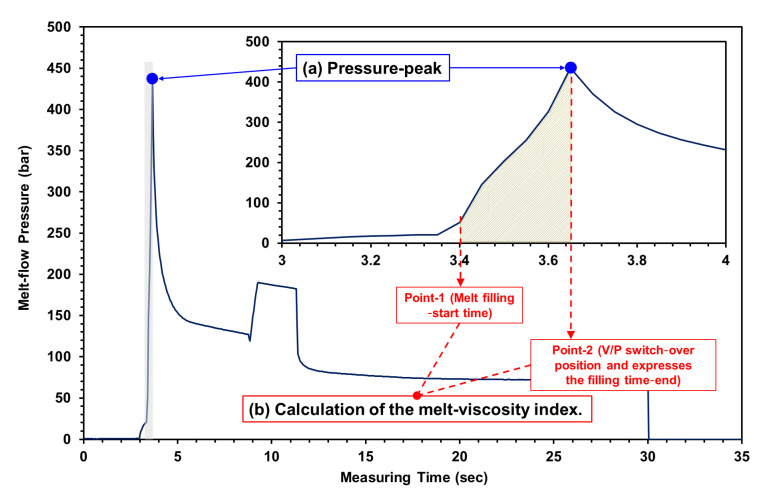
Measurement method of the melt flow pressure and melt-viscosity index: (**a**) definition of the melt flow pressure peak; (**b**) calculation of the melt-viscosity index.

In this study, an injection/foaming molding process involving a scientific melt pressure measurement was employed, and the plasticizing and molding parameters, melting pressure, and viscosity index were analyzed and investigated. In order to grasp the change in melt flow pressure during the injection molding process, the melt flow pressure was measured using the nozzle pressure sensor, and its pressure peak was measured at maximum pressure; the sensors were embedded in the nozzle tube and parallel to the inner tube wall (Figure 5; the nozzle melt pressure experiment was conducted using nozzle pressure sensors from the DYNISCO agent (PT4636-30M-3/18; Taichung City, Xitun District, Taiwan)). The experimental results of Kruppa and Holzinger [21] revealed that when using a pressure sensor at the nozzle, the pressure profiles during filling varied with the material, whereas the pressure profiles during packing remained unchanged. When using a pressure sensor in the cavity, pressure profiles during filling did not vary, whereas pressure profiles during packing varied noticeably. Based on differences between filling pressure profiles, we deduced that mounting a pressure sensor at the nozzle would be preferable; i.e., pressure measurements obtained in the cavity could be disregarded in calculating the VI. Figure 6 shows pressure profiles detected by a sensor during the filling stage of the injection molding process. For hydro-mechanically driven machines, this is carried out by measuring the driving hydraulic pressure. The pressure is converted to plastic melt pressure with the area ratio between the hydraulic cylinder and screw diameter. The position of the screw is measured by an appropriate external position sensor. An injection work equals the displacement of the molten plastic volume flows against a resistance. Similar to a viscometer, the work is measured during the injection phase as an integral of the injection pressure over the screw stroke. For integration over time, the result is injection energy instead of work. In contrast, the viscosity of the plasticized polymer melt is characterized by a defined key indicator in the injection phase. The corresponding process variable is to be measured in a suitable way; the melt-viscosity index was expected as an adaptive control feature value for future scientific injection molding and will be calculated by Equation (1) [23,24,25].

#### 2.3.2. Scan and Analysis for Inside the Foamed Parts

**Figure 7:**
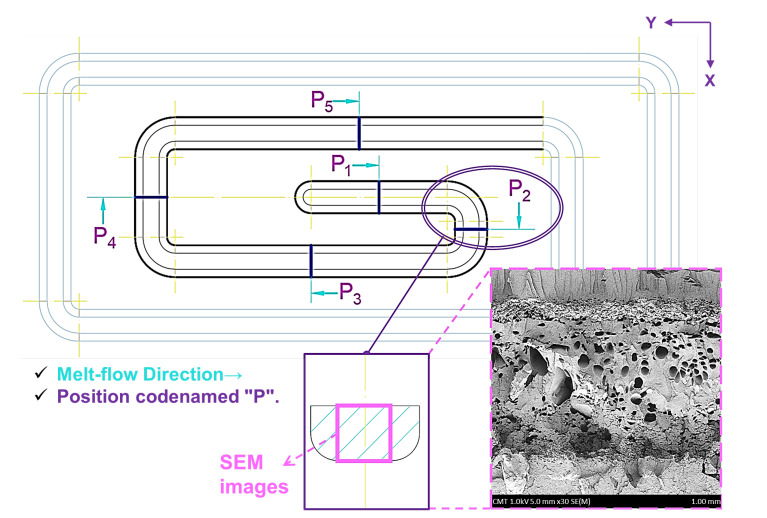
Schematic of scanning electron microscopy of average foam size after foaming in the melt flow specimens.

In the process of releasing gas into the barrel and mixing with the melt within, the inside of the barrel is a high-pressure/high-temperature and enclosed environment, so the compatibility between the two is determined by (i) the pressure and temperature inside the barrel on the phase change of the gas, (ii) the gas refining function released by the permeable steel at the front of the screw, and (iii) trend change for the pressure drop when the melt is actually injected. Therefore, when the gas-mixed melt is actually injected, the density change of its foaming structure is investigated through the flow path of the test piece and different cutting positions (Figure 7).

In contrast, through the obtained SEM, Equation (2) was imported to calculate the foaming characteristics [8]; the cell size, cell density, and expansion ratio are the three basic parameters characterizing cell structure. Among them, the definition of cell density ($N_{c}$) can be found as follows, and the “$N_{c}$” refers to the cell density, where “n” is the number of cells in a microscopic picture with the area “A” (in cm^2^).

(2) $$N_{c}={\left(\frac{n}{A}\right)}^{3/2}Ø$$

#### 2.3.3. Flexural Strength Test

**Figure 8:**
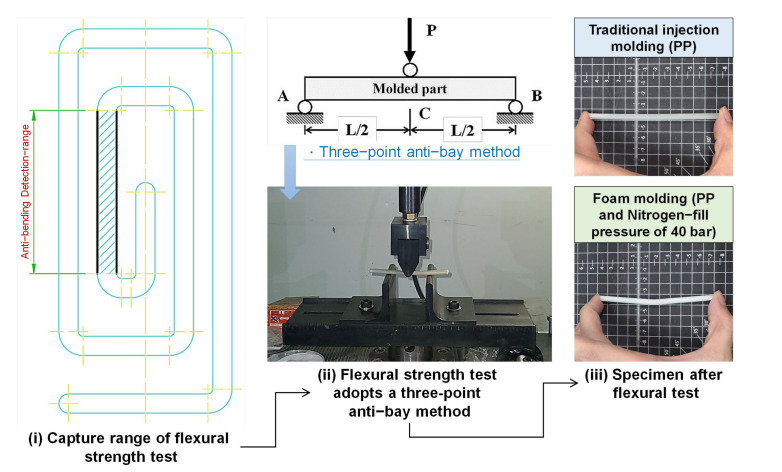
Measuring locations for the flexural strength test.

The flexural strength test was conducted on an AG-100 KN machine with full loads of 1000 kg/f, with an employed displacement rate of 10 mm/min. For each type of testing, ten samples molded under the same molding conditions were used (the cutting position of the specimen is shown in Figure 8 (i)), and the average value of the ten test specimens was used for analysis. Flexural test procedures followed the ASTM-D790 and SG323-81 standards (Figure 8 (ii)). Figure 8 (iii) is the flexural diagram of the traditional injection-molded PP specimen and the foamed injection PP specimen after the actual test.

## 3. Results

### 3.1. Effects of Nitrogen-Fill Content Variation and Melt Fluidity Properties

#### 3.1.1. Melting Pressure and Its Relevance to Viscosity Index

**Figure 9:**
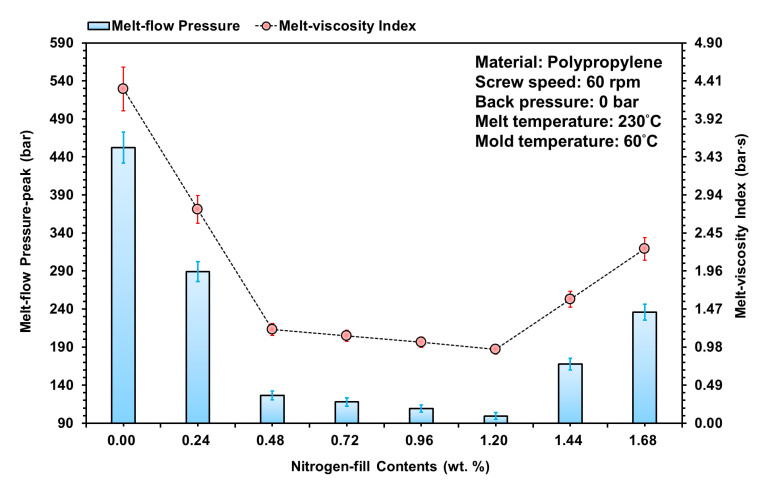
Influence of nitrogen fill content variations on melting pressure and melt-viscosity index for melt flow specimens.

The injection machine and the foaming technology developed from this study, and the actual injected molding experiment, used nitrogen as the source of gas to be used for foaming, with a melt-pressure-sensing component installed at the gate. Then, through different gas contents, the gas-mixed melt was investigated in terms of the pressure change during the plasticization to injection stage, and a formula with melt-viscosity index was introduced to compare the rheological behavior of the melt. Figure 9 is the melt flow pressure peak and the calculated melt-viscosity index under changing gas contents; it can be seen from the figure that as the gas content increases, the fluidity and viscosity properties of the material both show improvements. From references [23,24,25], it can be seen that the output of gas can reduce the viscosity property of the material, which relatively affects the fluidity of the raw material; the better the fluidity, the smaller the resistance, and the melt pressure is relatively lowered. This also reflects the mixing of the melt with gas during the plasticization stage, where the gas solubility increases in the high-temperature and high-pressure environments. After the gas is refined by the permeable steel, it can increase the specific surface area of the gas, which is equivalent to an increase in the refined solubility and mixability, and the melt compatibility also increases. It can also be compatible with the melt and improve its fluidity. Thus, the flow resistance of the melt decreases, and the melt-viscosity index calculated from the melt pressure also decreases.

In contrast, when gas content was set to higher than 1.44 wt. %, the melt caused intense foaming due to pressure release during the injection process; as the pressure sensor is relatively compressed, the captured pressure and melt-viscosity index of the melt is large, so the gas content will be greater than the preset range of 0.48~1.2 wt. %. Additionally, when gas content was set to 0.24 wt. %, the gas was compatible with the melt, the fluidity was improved, and the pressure peak value was reduced to 36%; whereas when it was set to within the 1.20 wt. % range, the pressure peak value was improved to between 72.1% and 77.9%, and the melt-viscosity index of the melt also decreased. From here, consequent studies shall focus on fluidity with content settings higher than 70%, which are the three gas contents of 0.48 wt. %, 0.96 wt. %, and 1.20 wt. %, to find the changes in the foaming type on the inside of the end product.

#### 3.1.2. Melt Fluidity and Its Effect on Foaming Characteristics

**Figure 10:**
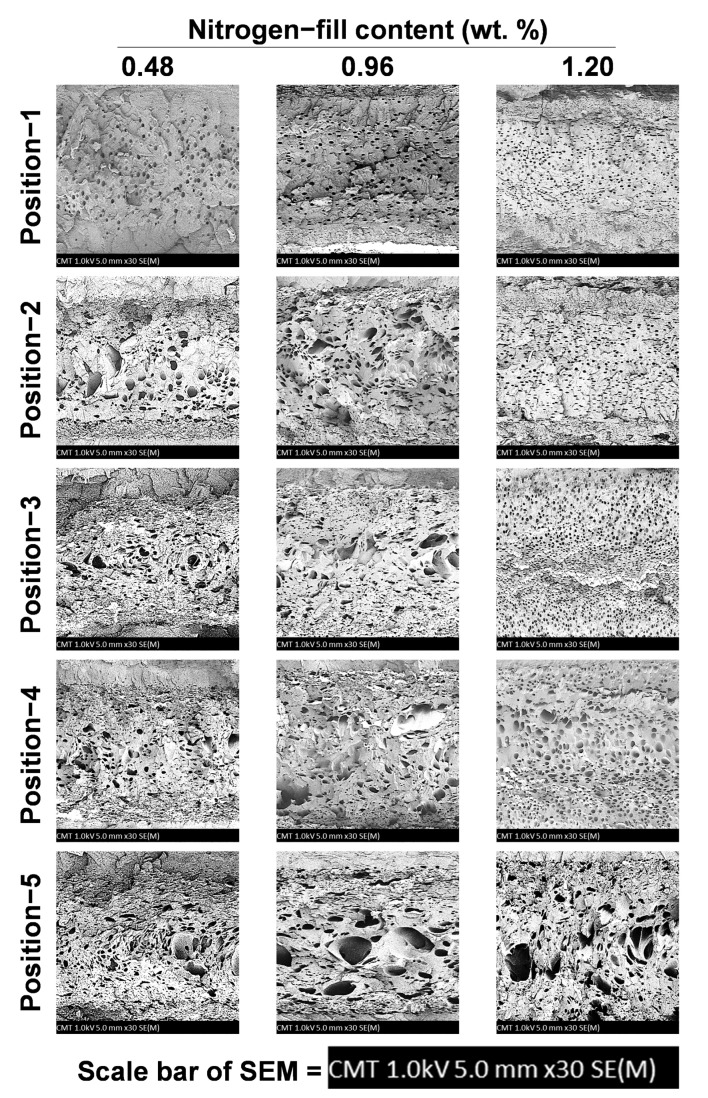
Scanning electron microscope image of structure distribution after foaming and its melt flow specimens at different positions (nitrogen fill contents of 0.48, 0.96, and 1.20 wt. %).

**Figure 11:**
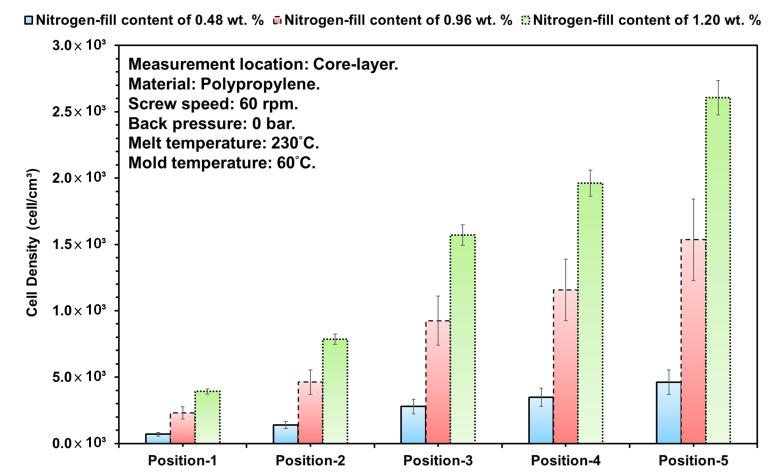
Calculated foaming morphology of all foaming and its melt flow specimens at different positions (nitrogen fill contents of 0.48, 0.96, and 1.20 wt. %).

Figure 10 shows the effects of foaming structure distribution on SEM for the three different gas contents mixed into the melt, where the gas and melt are made compatible by undergoing the plasticization process, and along the flow path of the melt during the injection stage and pressure-dropping trend. It can be seen from the figure that the growth of foam is dependent on the decreasing trend of pressure in the melt, and as the melt changes along the flow path, the foaming structure shown near the SEM cross-sectional area (Position 1) of the gate is the area of the melt larger than the cell density (Figure 11).

- As the melt passes the first bend in the test piece, the pushing pressure is easily inhibited by the impact of the bend. Therefore, the pressure after the first bend is unable to transmit, and the foaming structure and its size after the first bend will naturally become increasingly large. At the same time, due to the heat transfer of the mold runner, the melt on the superficial level will cure during the flowing stage in the melt-filling stage. Therefore, the thickness of the superficial level after Position 2 will gradually become more visible. In contrast, when gas content increases, it also means the gas generation ratio and density inside the melt are increased. Thus, although Position 1 is close to the gate, as the content is increased, the area of the foam will also gradually increase.
- Since the gas content is increased, the foaming structure and its density obtained will also gradually increase, followed by the flow path and decreasing pressure trend of the melt. Additionally, the pressure inside the melt equals the changes that affect the growth of foaming structure and density, and under the trend along the melt flow path in response to the changes of the pressure decrease, the ratio of foam growth has increased, whereas the melt undergoes the filling stage and is cooled/cured by the mold runner, and the ratio of the superficial level of the end product becomes increasingly clear. At the same time, as the gas content increases, the internal pressure of the foam growth is greater than the internal pressure of the melt, the foam structure increases, and the area of the superficial level reduces.

Based on the trend of the above melt pressure, melt-viscosity index, and the foaming structure and its density, the trends of all three were positively related; through the input of gas and increase of its content during the plasticization stage, it increased the lubricity/fluidity within the melt. The obtained melt was more easily pushed and did not require the screw to provide excessive pressure to push the melt into the mold cavities, and the foaming structure also changed. Therefore, consequent studies shall introduce parameter changes (screw speed, melt temperature, and injection speed) to find the effects of changes in the melt-viscosity index and the cell density of the melt; the observation position for foaming structure is Position 3, and using the bending strength test of the end product as the basis, 0.48 wt. % and 1.20 wt. % were selected from the gas content from the above study to define the changes in low/high gas content individually.

### 3.2. DOE and Its Correlation Analysis of Quality Characteristics

#### 3.2.1. Screw Speed Variations

**Figure 12:**
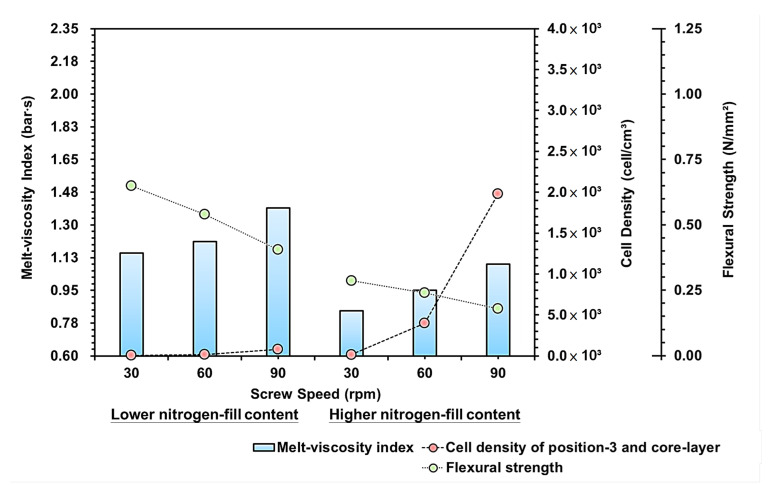
Variation of screw speeds and lower/higher nitrogen fill content on the melt-viscosity index, cell density, and flexural strength.

Figure 12 is the trend diagram for the effect of the melt-viscosity index, cell density, and bending strength on the screw rotation speed; the increase in screw speed significantly increased the flow rate, feeding speed of the material, plasticization time/temperature, and viscosity, etc. of the thermoplastic [31]. It can be seen from the figure that the screw rotation speed can directly affect the mixability of the plastic inside the barrel, and increase the flow rate and viscosity of the PP.

- Due to the stretching flow of the flow path change, the melt pressure under pressure will cause the melt-viscosity index to increase. To cooperate with the screw at low speed and the changes of low/high gas content, the plasticizing time is longer due to low rotation speed, which increases the compatibility between the gas and the melt during the plasticization stage. Therefore, under the setting of low rotation speed and increased gas content, the pressure, flow resistance, and melt-viscosity index during the filling process is relatively lower.
- Furthermore, within the changing screw speed, when the rotation speed increases, the refining effects of the gas increase as the gas is released into the melt through the permeable steel, making it easier to fuse into the melt. Therefore, it can be seen from the cell density trend that the melt-viscosity index and the cell density in the changing screw speed are negatively related.
- By comparing the strengths of the bending test, the cell density equals reducing the material density of the melt. Thus, the higher the cell density, the more the foaming structure and the bending resistance are reduced. So, with the increased screw speed and gas content, the bending strength resistance also decreases.

#### 3.2.2. Melt Temperature Variations

**Figure 13:**
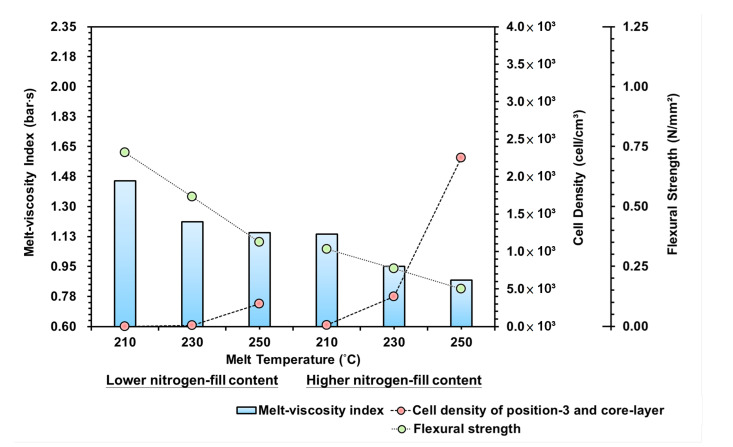
Variation of melt temperatures and lower/higher nitrogen fill content on the melt-viscosity index, cell density, and flexural strength.

Figure 13 is the trend diagram for the effect of the melt-viscosity index, cell density, and bending strength on the melt temperature. It can be seen from the diagram that the melt temperature is the temperature of the heating fin for the injection machine/plasticization unit, so the temperature of the melt will affect the retreat rate of the screw; a lower melt temperature setting will mean that the plastic inside the barrel cannot be moved easily, and the plasticization time will increase and the compatibility between the melt and the gas will be enhanced.

- Under low material temperature and increased gas content settings, the pressure, flow resistance, and melt-viscosity index during the filling stage were relatively lower. In contrast, the lower melt temperature affected the gas in the output and plasticization process, making it difficult to become compatible with the melt, thus yielding a lowered cell density.
- At the same time, in response to the lower melt temperature, the cooling/curing efficacy of the mold runner increased, leading to a higher ratio in the melt curing area than foam growth. Conversely, with the higher melt temperature, a longer cooling time in the mold runner was required for curing, during which the foam continued to grow within the high-temperature melt in the core level, thus increasing the cell density. It can be seen that the melt-viscosity index and the cell density in the changing melt temperature are positively related.
- By comparing the strengths of the bending test, the cell density equals reducing the material density of the melt. Thus, the higher the cell density, the more the foaming structure and the bending resistance are reduced. Therefore, with the increased melt temperature and gas content, the bending strength resistance also decreases.

#### 3.2.3. Injection Speed Variations

**Figure 14:**
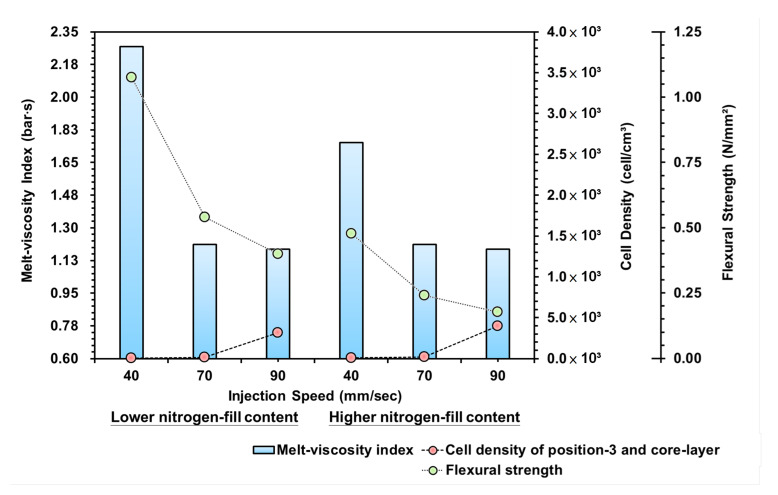
Variation of injection speeds and lower/higher nitrogen fill content on the melt-viscosity index, cell density, and flexural strength.

Figure 14 is the trend diagram for the effect of the melt-viscosity index, cell density, and bending strength on the injection speed. It can be seen from the diagram that the injection speed is the set value for the screw propulsion speed of the injection machine during the injection stage; the faster the screw propulsion speed, the shorter the time for the melt to enter the mold cavity, which means that the melt will remain inside the mold cavity for a longer time. Therefore, for the changing injection speed, the melt-viscosity index obtained from a lower injection speed is larger, which is due to the melt propulsion speed being slower.

- The melt inside the mold produced high shear force due to the cooling/curing effect of the mold runner, which increased the viscosity of the melt. However, when using the faster injection speed, the curing efficacy of the mold runner was unable to keep up with the propulsion speed to push the melt into the mold cavity, so the pressure, flow resistance, and viscosity factor during the filling stage were relatively lower.
- In contrast, since the slower injection speed caused a greater melt viscosity factor inside the mold, this means that there was greater pressure within the melt, which affected the growth ratio of the foam. Thus, when representing the cell density, it was found that the trend for the cell density between the injection speed of 40 and 70 mm/s range was not visible. Conversely, the faster injection speed increased the time the melt remained inside the mold, and the pressure within the melt was lower, thus increasing foam growth and cell density. It can be found from the trend that the viscosity factor and the cell density in the changing injection speed are positively related.
- By comparing the strengths of the bending test, the cell density equals reducing the material density of the melt. Thus, the higher the cell density, the more the foaming structure and the bending resistance are reduced. Therefore, with the increased injection speed and gas content, the bending strength resistance also decreases.

## 4. Conclusions

A structural-foam injection machine (with nitrogen supply equipment connected to a unique plasticizing mechanism) was employed to determine specimens’ melt-rheology quality and after-foam structures in injection-molding experiments under different N2-fill contents and process parameters. The notable research results are as follows:

i. Through the installation of the real-time sensor system for the pressure at the gate, the pressure history of the plasticization/injection/foaming process and the changing trend of the melt-viscosity index could be successfully determined. Additionally, in the pressure trend of the gas–melt mixture, the amount of the gas content could be confirmed, which is an important factor that indirectly affects the flow feature of the melt filling.
ii. The trend between the melt pressure and the obtained melt-viscosity index was calculated to be positively related. Additionally, in the trend for different gas contents, the gas content could affect the fluidity of the material when within a set range. However, if the content exceeded the range, the pressure trend was unavailable.
iii. During the process of foam molding, the melt-viscosity index and the foaming structure and its density were positively related; through the input of gas and an increase in its content during the plasticization stage, it increased the lubricity/fluidity within the melt. The obtained melt was more easily pushed and did not require the screw to provide excessive pressure to push the melt into the mold cavities. The foaming structure also changed.
iv. The higher the cell density, the more the bending resistance decreased, which also reduced the bending strength because of the interaction between the cell density, the thickness of the skin layer, and the foaming structure, leading to changes in its strength.

## Figures and Tables

**Table 1:** Configuration of the process parameters for the molding of specimens.

Constant Factors		Controllable Factors			
Clamping Force (ton)	50	Screw Speed (rpm)	30	60	90
Mold Temperature (°C)	60	Melt Temperature (°C)	210	230	250
Cooling Time (s)	40	Injection Speed (%)	40	70	90
Injection Pressure (bar)	140				
Injection Time (s)	1.5				
V/P Switch-over Position (mm)	1				
Back Pressure (bar)	0				
Gas-fill Time (s)	10				

**Table 2:** Random order of the treatments for molding of the specimens with lower/higher nitrogen-fill content.

Exp.	Screw Speed (rpm)	Melt Temperature (°C)	Injection Speed (mm/s)
1	30	230	70
2	60	230	70
3	90	230	70
4	60	210	70
5	60	230	70
6	60	250	70
7	60	230	40
8	60	230	70
9	60	230	90

## Data Availability

The data presented in this study are available upon request from the corresponding author.
